# pH-Responsive Alginate-Based Microparticles for Colon-Targeted Delivery of Pure Cyclosporine A Crystals to Treat Ulcerative Colitis

**DOI:** 10.3390/pharmaceutics13091412

**Published:** 2021-09-06

**Authors:** Murtada A. Oshi, Juho Lee, Jihyun Kim, Nurhasni Hasan, Eunok Im, Yunjin Jung, Jin-Wook Yoo

**Affiliations:** 1 Faculty of Pharmacy, Omdurman Islamic University, Omdurman 14415, Sudan; oshiphar@yahoo.com; 2 College of Pharmacy, Pusan National University, Busan 46241, Korea; jhlee2350@gmail.com (J.L.); jihyun@pusan.ac.kr (J.K.); hasni1986.nh@gmail.com (N.H.); eoim@pusan.ac.kr (E.I.); jungy@pusan.ac.kr (Y.J.)

**Keywords:** cyclosporine A, colon-targeted delivery, ionic gelation, microparticles, ulcerative colitis

## Abstract

Cyclosporine A (CsA) is a potent immunosuppressant for treating ulcerative colitis (UC). However, owing to severe systemic side effects, CsA application in UC therapy remains limited. Herein, a colon-targeted drug delivery system consisting of CsA crystals (CsAc)-loaded, Eudragit S 100 (ES)-coated alginate microparticles (CsAc-EAMPs) was established to minimize systemic side effects and enhance the therapeutic efficacy of CsA. Homogeneously-sized CsAs (3.1 ± 0.9 μm) were prepared by anti-solvent precipitation, followed by the fabrication of 47.1 ± 6.5 μm-sized CsAc-EAMPs via ionic gelation and ES coating. CsAc-EAMPs exhibited a high drug loading capacity (48 ± 5%) and a CsA encapsulation efficacy of 77 ± 9%. The in vitro drug release study revealed that CsA release from CsAc-EAMPs was suppressed under conditions simulating the stomach and small intestine, resulting in minimized systemic absorption and side effects. Following exposure to the simulated colon conditions, along with ES dissolution and disintegration of alginate microparticles, CsA was released from CsAc-EAMPs, exhibiting a sustained-release profile for up to 24 h after administration. Given the effective colonic delivery of CsA molecules, CsAc-EAMPs conferred enhanced anti-inflammatory activity in mouse model of dextran sulfate sodium (DSS)-induced colitis. These findings suggest that CsAc-EAMPs is a promising drug delivery system for treating UC.

## 1. Introduction

Ulcerative colitis (UC) is a chronic, relapsing disease of the gastrointestinal tract (GIT) that causes inflammation and ulcers in the mucosal lining of the colon and rectum [1]. This multifactorial disease is associated with dysregulated expression of multiple genes in combination with microbial and environmental factors. UC causes a variety of symptoms, such as fever, abdominal cramping, and frequent diarrhea with rectal bleeding. The natural course of UC comprises quiescent phases interrupted by relapse [2,3]. Currently, UC therapy involves the use of anti-inflammatory drugs, such as aminosalicylates, glucocorticoids, and immunosuppressants [4,5]. Among them, cyclosporine A (CsA), a peptide produced by the fermentation by aerobic fungi such as *Aspergillus terreus* and *Tolypocladium inflatum,* is widely employed as an immunosuppressant owing to its potent anti-inflammatory effects [6]. Notably, it exerts its anti-inflammatory activity via inhibiting interleukin-2 production in activated T-lymphocytes through a calcineurin-dependent pathway, thereby alleviating inflammation [7]. However, despite its robust therapeutic efficacy, the use of CsA is largely limited owing to its toxicity and distressing systemic side effects, such as hypertension, seizures, nephrotoxicity, renal dysfunction, and opportunistic infections [8,9]. In addition, as CsA is distributed throughout the body, only a small amount of drug molecules can accumulate at the target site (inflamed regions of the colon), resulting in a reduced anti-inflammatory effect [10]. Therefore, to avoid systemic side effects and increase the therapeutic efficacy, a colon-targeted drug delivery system that could effectively deliver an appropriate amount of CsA molecules to the colitis affected regions without systemic absorption needs to be established [11,12,13].

As colon-targeted delivery systems, several CsA-loaded polymeric nano- and microparticles have been developed to minimize systemic absorption of the drug and enhance drug accumulation in inflamed colonic tissues [10,11,12,14]. In these drug delivery systems, CsA incorporation into a polymeric matrix, such as poly(lactic-*co*-glycolic acid) and poly methacrylate-based copolymers, prevents the initial burst release of CsA in the stomach and small intestine, thus resulting in reduced systemic absorption. However, although these systems allow the successful delivery of CsA to the colon during UC therapy, intrinsic limitations persist, including low drug loading capacity, complicated fabrication processes, low cost-effectiveness, and excipient-associated toxicities [15,16]. In particular, the low drug loading capacity is a major challenge, as it may result in insufficient drug delivery to the colon during UC therapy. In this regard, pure drug crystals with a particle diameter ˂10 μm could be a promising drug delivery system to overcome these limitations [17,18]. Unlike polymeric nano- and microparticles, pure drug crystals can deliver an extremely high amount of drugs to the colon owing to their high loading capacity (~100%) [19]. In addition, pure drug crystals could benefit from the enhanced permeability effect induced by inflammation and accumulate in inflamed colonic regions [20,21]. Accordingly, delivering pure CsA drug crystals to the colon would be a desirable strategy for treating UC.

As an attractive drug carrier for the CsA crystal delivery system, alginate-based microparticles have gained momentum given the favorable characteristics of alginate, including low toxicity, biodegradability, relatively low cost, and gel-forming ability [22]. In addition, especially for alginate microparticles fabricated by ionic gelation, suitable carriers are available for encapsulating nano- or micro-sized drug particles, proteins, and probiotics. The sugar groups of alginate in polymer networks effectively stabilize the suspended cargos, and the surrounding alginate on microparticle surfaces act as a protective barrier against various external environment stressors [23,24]. Based on these attributes, various types of alginate-based microparticles have been developed as drug carriers for treating UC. However, only limited success has been achieved using alginate microparticles due to uncontrolled initial drug release in the stomach and small intestine, resulting in drug loss before reaching the colon [25,26]. To overcome these limitations, coating alginate-based microparticles with pH-sensitive polymers, such as Eudragit S 100 (ES), is a desirable strategy [27]. As ES remains insoluble at a pH below 7, surface coating with ES could prevent drug leakage in the stomach and small intestine [20]. In contrast, after exposure to the colonic environment (pH > 7), the release of the incorporated drug from alginate-based microparticles can be triggered by the dissolution of the ES layer and disintegration of alginate microparticles. For these reasons, the coating of alginate microparticles with ES could efficiently deliver CsA crystals to the colon.

In the present study, we developed CsA crystals-loaded, ES-coated alginate microparticles (CsAc-EAMPs) as a colon-targeted drug delivery system to enhance the therapeutic efficacy and reduce systemic side effects of CsA during UC therapy. After characterization of CsAc-EAMPs, the drug release profile was determined in pH-changing media. Furthermore, the enhanced in vivo therapeutic efficacy of CsAc-EAMPs was evaluated in a dextran sulfate sodium (DSS)-induced colitis mouse model.

## 2. Materials and Methods

### 2.1. Materials

CsA, chitosan (molecular weight [MW] 50,000–190,000 Da, viscosity 20–30 cP, and deacetylation ≥75%), sodium alginate (MW 80,000–120,000 Da, viscosity ≥2000 cP, and mannuronate/guluronate ratio 1.56), and Mayer’s hematoxylin solution were purchased from Sigma-Aldrich (St. Louis, MO, USA). ES (MW ~125,000) was generously gifted by Evonik (Essen, Germany). DSS (MW 36,000–50,000) was obtained from MP Biomedicals (Irvine, CA, USA). Eosin-Y solution was purchased from Daejung Chemicals & Metals (Shiheung, Korea). Primary anti-E-cadherin was purchased from BD Bioscience (San Jose, CA, USA). Alexa Fluor 488-conjugated AffiniPure goat anti-mouse IgG was purchased from Jackson ImmunoResearch Laboratories (West Grove, PA, USA). All other chemicals, reagents, and solvents were of the highest commercially available purity grade.

### 2.2. Preparation of CsAc-EAMPs

#### 2.2.1. Preparation of CsAc

**Figure 1:**
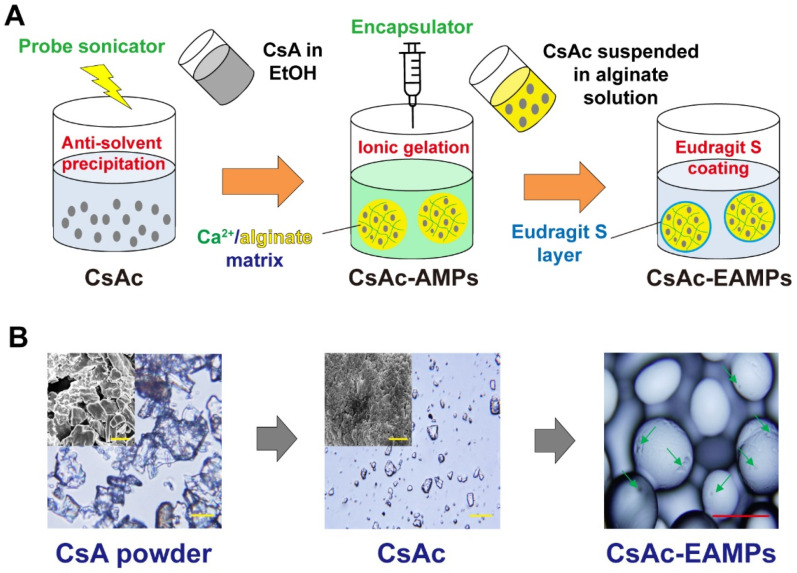
(**A**) Schematic illustration of CsAc, CsAc-AMPs, and CsAc-EAMPs fabrication using three sequential processes: fabrication of CsAc, incorporation of CsAc into the alginate microparticles, and ES coating of the microparticles. (**B**) SEM and microscopic images of CsA powder, CsAc, and CsAc-EAMPs. Green arrows represent CsAc loaded in CsAc-EAMPs. Yellow and red scale bars represent 10 μm and 50 μm, respectively. CsAc, cyclosporine A crystals; CsAc-AMPs, CsA crystal-loaded alginate microparticles; CsAc-EAMPs, CsA crystal-loaded, Eudragit S 100 coated alginate microparticles; SEM, scanning electron microscopy.

CsA crystals (CsAc) were prepared by employing an anti-solvent precipitation method, using sonication and ethanol as a water-miscible solvent (Figure 1). In brief, 2 mg CsA powder was dissolved in 5 mL ethanol and filtered through a 0.45 µm syringe filter to obtain a clear drug solution. The resulting drug solution was injected at a rate of 0.5 mL/min into 20 mL distilled water, sonicated at 90 W for 5 min at 4 °C and stirred. The solid drug particles were precipitated immediately upon mixing the two liquids. After stirring for 20 min, a precipitated CsA suspension was obtained, collected by centrifugation at 20,000× *g* for 10 min, and washed three times with distilled water to remove ethanol.

#### 2.2.2. Preparation of CsAc-EAMPs

CsAc-EAMPs were fabricated via two sequential processes: fabrication of CsAc-loaded alginate microparticles (CsAc-AMPs), followed by enteric-coating of the particle surface using chitosan and ES. First, CsAc-AMPs were prepared using an ionic gelation method with an encapsulator (Encapsulator B-390/B-395 Pro, Buchi, Flawil, Switzerland). Figure 1A shows the scheme for microencapsulation of CsAc within sodium alginate microparticles using calcium chloride as a cross-linker. In brief, 10 mg CsAc was well dispersed in 20 mL freshly prepared sodium alginate solution to form a suspension. Then, a 0.5% (*w*/*v*) calcium chloride solution was freshly prepared as a cross-linker. The above suspension was filled in the encapsulator and injected into the solution (nozzle size: 80 µm, flow rate: 1.1 mL/min, frequency: 1300 Hz), thereby forming CsAc-AMPs after 1 h. For preparing CsAc-EAMPs, CsAc-AMPs were coated with chitosan and ES using physical electrostatic interactions as a coating mechanism. Briefly, CsAc-AMPs were introduced into 2 mg/mL of chitosan in 0.1 M acetic acid solution. After stirring for 30 min at 4 °C, the CsAc-AMPs were recovered by centrifugation (2000× *g* for 10 min). Then, the microparticles were suspended in an ES solution (2 mg/mL, pH 7.4) for 30 min at room temperature. After incubation, CsAc-AMPs were obtained by centrifugation (2000× *g* for 10 min).

#### 2.2.3. Characterization of CsAc-EAMPs

The shape of prepared CsAc was analyzed using scanning electron microscopy (SEM, Supra 40, Carl Zeiss, Jena, Germany). Raw CsA powder and CsAc were suspended in water, dropped on a carbon tape, and air-dried at room temperature before visualization by SEM at an acceleration voltage of 1–5 kV. The particle shape of the CsAc-EAMPs was analyzed by light microscopy (BX53, Olympus, Tokyo, Japan). A specific amount of CsAc-EAMPs was placed on a glass slide and covered prior to imaging. The particle size of CsAc was determined using ImageJ software (National Institutes of Health, Bethesda, MA, USA) [28,29].

#### 2.2.4. Determination of CsA in CsAc-EAMPs

The amount of CsA in CsAc-EAMPs was determined using high-performance liquid chromatography (HPLC) (Shimadzu, Tokyo, Japan). Prior to HPLC analysis, CsAc-AMPs and CsAc-EAMPs were placed in vials containing ethanol and shaken vigorously at room temperature for 24 h for the complete extraction of CsA from microparticles. The samples were centrifuged, and the supernatants were filtered through a 0.22-μm filter. The HPLC analysis conditions were as follows: detection wavelength, 210 nm; mobile phase, a mixture of acetonitrile and water (75:25); column, VDSpher 100 C18-E column (4.6 mm × 150 mm, 3.5 μm, VDS Optilab, Berlin, Germany); injection volume, 20 μL; flow rate, 1.5 mL/min; oven temperature, 65 °C [30]. Encapsulation efficiency (EE) and drug loading (DL) were calculated from three different batches in each group using the following Equations (1) and (2):

(1) $$\mathrm{EE}\%=\frac{\mathrm{Weight}\;\mathrm{of}\;\mathrm{CsAc-EAMPs}\;}{\mathrm{Theoretical}\;\mathrm{weight}\;\mathrm{of}\;\mathrm{CsAc-EAMPs}\;}\;\times \;100$$

(2) $$\mathrm{DL}\%=\frac{\mathrm{Weight}\;\mathrm{of}\;\mathrm{drug}\;\mathrm{in}\;\mathrm{CsAc-EAMPs}\;}{\mathrm{Weight}\;\mathrm{of}\;\mathrm{CsAc-EAMPs}\;}\;\times \;100$$

### 2.3. Drug Release Study in pH-Changing Media

The pH-dependent drug release profiles of CsAc, CsAc-AMPs, and CsAc-EAMPs were examined using the dialysis bag diffusion method, with some modifications [31,32]. In brief, dialysis membranes (MW cut-off, 12,000–14,000; Servia Electrophoresis GmbH, Heidelberg, Germany) were soaked overnight in distilled water for complete wetting before use. The membranes were then filled with 5 mL suspension of CsAc, CsAc-AMPs, and CsAc-EAMPs, placed in the release medium containing 50 mL solution with gradually changing pH from 1.2 to 6.8 to 7.4, to mimic the pH of the stomach, small intestine, and colon, respectively. The experiments were performed at 37 °C with a stirring speed of 50 rpm. A 1000 μL sample was removed from the medium at predetermined time intervals, centrifuged at 25,000× *g* for 20 min, and the drug content in the supernatants was analyzed using HPLC with the method mentioned above.

### 2.4. In Vivo Therapeutic Efficacy of CsAc-EAMPs

#### 2.4.1. Colitis Induction and Drug Treatment Protocol

The animal study was conducted according to the guidelines of the Declaration of Helsinki and approved by the Institutional Review Board (or Ethics Committee) of Pusan National University Institutional Animal Care and Use Committee (protocol code PNU-2019-2420 on 23 October 2019). DSS-induced colitis was induced in mice using a previously reported method with some modifications [19]. Briefly, ICR mice (male, 6-week-old) were obtained from Samtako Bio Korea (Osan, Korea). Mice were maintained in isolated cages under a 12 h light/dark cycle with controlled humidity and temperature (25 °C). Food and water were freely available during acclimatization. After a week, mice were treated with DSS (2.5% *w*/*v*) in water provided ad libitum for seven days to induce colitis. Age-matched male ICR mice provided with normal tap water served as the healthy control group. After colitis induction, CsA formulations (CsAc, CsAc-AMPs, and CsAc-EAMPs) were daily administered orally by using a flexible plastic oral gavage at dose of 15 mg/kg for seven consecutive days.

#### 2.4.2. Macroscopic Assessment of Colitis

For assessing the therapeutic effects of CsAc-EAMPs, the disease activity index (DAI) of mice was calculated based on changes in body weight, stool state, and rectal bleeding, recorded from day 1 to day 14 of the experiment [33]. In brief, the change in body weight was scored on a scale of 0 to 4 as follows: no weight loss, 0; 1–5% weight loss, 1; 5–10%, 2; 10–20%, 3; >20%, 4. For stool consistency, 0 points were given for well-formed stool, 2 for pasty stool, and 4 for liquid stool. Bleeding was scored as 0 for no blood, 2 for less bleeding, and 4 for severe bleeding. The mean of these scores formed the DAI, ranging from 0 (healthy) to 4 (maximal colitis). On the last day of the experiment, all the mice were sacrificed for further investigations. Colon length was measured after excising the large intestine (from the cecum to the anus) as an indicator of colitis severity.

#### 2.4.3. Endoscopic Analysis of Colitis

For direct monitoring of colitis severity in live mice, endoscopic analysis was performed using a Veterinary Endoscopy System (Hyunjoo In-Tech, Seoul, Korea), following the manufacturer’s instructions. Briefly, on the last day of the experiment, mice were anesthetized with isoflurane, and the colon was visualized after air inflation of the colon. For quantitative analysis of the colitis severity, the murine endoscopic index of colitis severity (MEICS) was used as previously described [34]. The score was calculated from the summation of individual scores of five parameters: thickening of the colon, stool consistency, presence of fibrin, granularity of the mucosal surface, and changes in vascular pattern. The scoring was conducted using the following criteria: thickening of the colon (0 points for transparent, 1 point for moderate, 2 points for marked, and 4 points for non-transparent bowel wall), stool consistency (0 points for solid stool, 1 point for soft but still shaped stool, 2 points for unshaped stool, and 3 points for spread stool), presence of fibrin (0 points for none, 1 point for detectable visible fibrin, 2 points for marked visible fibrin, and 3 points for abundantly visible fibrin), the granularity of the mucosal surface (0 points for none, 1 point for moderate, 2 points for marked, and 3 points for extreme granularity of the mucosal surface), and changes in vascular pattern (1 point for moderate change, 2 points for marked change, and 3 points for bleeding).

#### 2.4.4. Histological Assessment of Colitis

For histological analysis, paraffin-embedded tissue samples were prepared following a previously reported method with some modifications [35,36]. Briefly, excised colon sections from each group were embedded in paraffin blocks after 24 h incubation in phosphate-buffered 10% formalin solution. After sectioning into 5 µm-thick slices, the samples were stained with hematoxylin and eosin (H&E) following the manufacturer’s protocol. The H&E-stained colon tissues from each group were observed and imaged using a light microscope (BX53, Olympus, Tokyo, Japan). For the semi-quantitative evaluation of colitis severity, each slide was graded following a previous method [37]. The grading criteria were as follows: 0, healthy; 1, slightly damaged; 2, presence pathological signs; 3, diffuse lesions; 4, significantly intensive changes; 5, totally disrupted colon tissues. After grading, the histological score was calculated by summing all scores of each group.

#### 2.4.5. E-Cadherin Immunostaining

In order to evaluate the E-cadherin expression in each colon sample, immunofluorescence staining was performed as previously reported [38]. Briefly, 5 µm-thick paraffin-embedded tissue sections were deparaffinized and incubated with 5% bovine serum albumin in phosphate-buffered saline (PBS) at room temperature for 1 h to block nonspecific antibody binding. After washing, the sections were incubated with anti-E-cadherin antibody (1:100 dilution in PBS at 4 °C for 12 h). The samples were then washed with PBS and incubated with Alexa Fluor 488-conjugated anti-rabbit secondary antibody (1:200 dilution) for 2 h at room temperature, followed by counterstaining with 4′,6-diamidino-2-phenylindole (DAPI). Confocal laser scanning microscopy (CLSM) images were obtained using an FV10i FLUOVIEW confocal microscope (Olympus, Tokyo, Japan).

#### 2.4.6. Myeloperoxidase (MPO) Activity Measurement

MPO activity in colon tissues was measured following a previously reported method [39]. Briefly, colon tissue samples (100 mg) were homogenized in 4 mL potassium phosphate buffer (50 mM, pH 6.0) containing 0.5% hexadecyltrimethylammonium bromide. After centrifugation (13,400× *g* at 4 °C for 6 min), 7 µL supernatant was added to 200 µL of 0.167% (*w*/*v*) mg/mL o-dianisidine hydrochloride with 0.0005% (*v*/*v*) hydrogen peroxide in a 96-well plate. The absorbance of each sample was measured at 450 nm using a microplate reader (iMark Microplate Reader, Bio-Rad Laboratories, Inc., Richmond, CA, USA).

### 2.5. Statistical Analysis

Data were analyzed using GraphPad Prism version 5.0 for Windows (GraphPad Software Inc., La Jolla, CA, USA). Two-tailed *t*-test, one-way ANOVA, or two-way ANOVA were performed to assess differences between groups. Statistical significance was set at *p* ≤ 0.05.

## 3. Results

### 3.1. Preparation and Characterization of CsAc-EAMPs

CsAc-EAMPs were successfully fabricated by employing three sequential processes: fabrication of CsAc, incorporation of CsAc into alginate microparticles, and ES coating on the microparticle surface (Figure 1A). First, to prepare homogeneous pure CsA microcrystals, a bottom-up crystallization method (anti-solvent precipitation method) was used combined with probe sonication. Before the micronization process, the CsA powder was predominantly irregular in shape, with particle sizes ranging from 10 to 20 µm (Figure 1A). However, the sonication-assisted micronization process resulted in homogeneous and well-dispersed CsAc, with a mean particle size of 3.1 ± 0.9 μm (Table 1, Figure 1B).

After fabrication of CsAc, CsAc-AMPs were fabricated via ionic gelation of alginate in the presence of calcium ions using an encapsulator. Globular-shaped, 46.4 ± 8.8 µm-sized CsAc-AMPs were obtained and exhibited 49 ± 9% CsA loading and 78 ± 7% EE (Table 1). To fabricate CsAc-EAMPs, ES was coated onto the surface and subsequently, 47.1 ± 6.5 µm-sized CsAc-EAMPs were obtained, demonstrating 48 ± 5% CsA loading and 77 ± 9% EE (Table 1). After coating with ES, CsA loading of CsAc-EAMPs moderately decreased compared with that of CsAc-AMPs; this may be due to the unwanted release of CsA during the coating process. In addition, owing to the high CsA loading in the microparticles, CsAc was observed on the surface of CsAc-EAMP microparticles (Figure 1B). 

The results are expressed as mean ± standard deviation (*n* = 100 for particle size analysis, *n* = 3 for encapsulation efficacy and drug loading measurement). CsAc, cyclosporine A crystals; CsAc-AMPs, CsA crystal-loaded alginate microparticles; CsAc-EAMPs, CsA crystal-loaded, Eudragit S 100 coated alginate microparticles.

### 3.2. Drug Release Study

**Figure 2:**
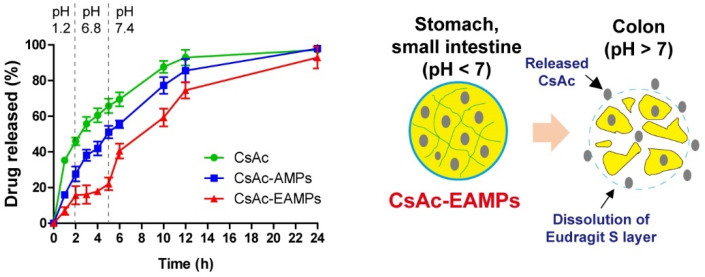
Drug release profiles of CsAc, CsAc-AMPs, and CsAc-EPMPs in media under different pH conditions and the proposed pH-dependent drug release mechanism of CsAc-EAMPs. Data are expressed as means ± standard deviation (*n* = 3). CsAc, cyclosporine A crystals; CsAc-AMPs, CsA crystal-loaded alginate microparticles; CsAc-EAMPs, CsA crystal-loaded, Eudragit S 100 coated alginate microparticles.

We examined the drug release profiles of CsA formulations by performing an in vitro drug release study using a pH-changing medium that simulated the pH conditions of the GIT. As shown in Figure 2, CsAc exhibited an initial burst drug release under the simulated pH condition of the stomach (pH 1.2), with over 40% of the total drug released during the first 2 h. After changing the pH of the medium to 6.8 to simulate the small intestinal pH, the release profile of CsAc was unaltered, and approximately 70% of the total drug was released in 5 h. After changing the pH to 7.4, more than 90% of the total drug was released from CsAc in 12 h. In the simulated pH of the stomach (pH 1.2), the initial burst release from CsAc-AMPs was slightly reduced when compared with that from CsAc, and approximately 30% of the total drug was released in 2 h. However, on continuing the experiment for 5 h, more than 50% of the drug was released under pH conditions simulating the small intestine (pH 6.8). Due to the porous structure of alginate microparticles, CsAc-AMPs failed to afford effective enteric protection to prevent premature drug release before reaching the colon. In contrast, the ES-coated alginate microparticles of CsAc-EAMPs acted as a protective layer, and less than 20% of the total drug was released during the first 5 h under pH 1.2 and 6.8. After exposure to the colonic pH condition (pH 7.4), the drug release rate increased as ES dissolved, and approximately 90% of the total drug was released from CsAc-EAMPs for up to 24 h. Collectively, these results demonstrated that CsAc-EAMPs conferred enteric protection to the drug and allowed colon-targeted delivery, which are desirable characteristics for treating UC.

### 3.3. In Vivo Therapeutic Efficacy of CsAc-EAMPs

#### 3.3.1. Macroscopic Assessment of Colitis

**Figure 3:**
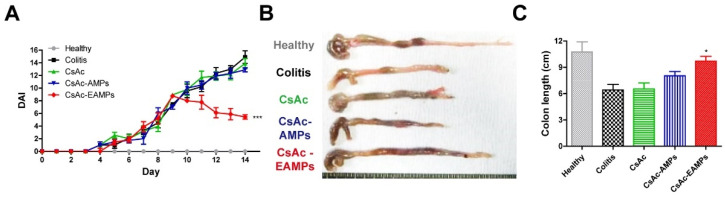
Macroscopic evaluation of colitis. (**A**) Changing profiles of disease activity index (DAI). (**B**) Representative colon image on day 14 (last day of the experiment). (**C**) Colon length. * and *** denote *p* < 0.05 and *p* < 0.001 compared to the colitis group. The results are expressed as means ± standard deviation (*n* = 8). CsAc, cyclosporine A crystals; CsAc-AMPs, CsA crystal-loaded alginate microparticles; CsAc-EAMPs, CsA crystal-loaded, Eudragit S 100 coated alginate microparticles.

The enhanced anti-inflammatory effects of CsAc-EAMPs were evaluated in a DSS-induced colitis mouse model. During the experiment, the DAI score representing the severity of colitis symptoms, including diarrhea, rectal bleeding, and body weight loss, was monitored to evaluate the therapeutic efficacy of formulated CsA formulations (Figure 3A). Following the administration of 2.5% DSS solution, all mice showed increasing DAI values from days 0 to 7, indicating successful induction of colitis. From day 7, DSS administration was stopped, and CsA formulations (15 mg/kg) were administered via oral gavage. From day 9 (2 days after treatment initiation), the DAI score decreased in the CsAc-EAMP-treated group, while other groups showed increasing DAI values. The DAI score continued to decrease with CsAc-EAMPs treatment, indicating that CsAc-EAMPs could ameliorate colitis symptoms by effectively modulating inflammatory reactions in the colon. In contrast, treatment with CsAc and CsAc-AMPs failed to reduce the DAI significantly when compared with the untreated colitis group, as insufficient CsA was delivered into the colon, which resulted from the failure of the colon-targeted CsA delivery.

On the last day of the experiment (day 14), all mice were sacrificed; then, the colons were excised to measure colon length as a parameter of colitis severity (Figure 3B,C). Owing to severe inflammation, the untreated colitis group exhibited a shorter colon length than that in the healthy group. In addition, the colon length of the CsAc- and CsAc-AMP-treated groups was similar to that of the colitis group, given the presence of severe inflammation. Conversely, the colon length was significantly increased in the CsAc-EAMP-treated group when compared with that of the untreated colitis group, indicating that the colons of the CsAc-EAMP-treated group recovered following the effective mitigation of colonic inflammation.

#### 3.3.2. Endoscopic Analysis of Colitis

**Figure 4:**
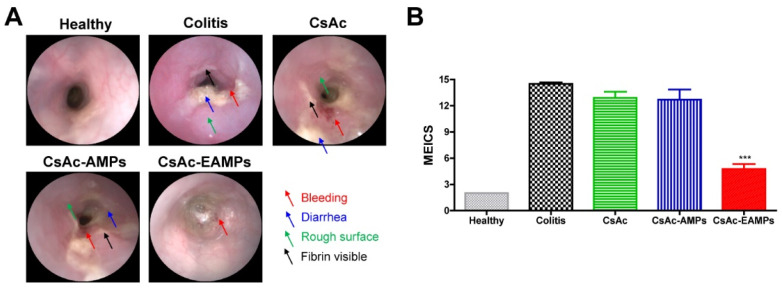
Endoscopic examination of the colon on day 14. (**A**) Representative endoscopic images (**B**) Murine endoscopic index of colitis severity (MEICS) scores. *** denotes *p* < 0.001 compared with the untreated colitis group. The results are expressed as means ± standard deviation (*n* = 8). CsAc, cyclosporine A crystals; CsAc-AMPs, CsA crystal-loaded alginate microparticles; CsAc-EAMPs, CsA crystal-loaded, Eudragit S 100 coated alginate microparticles.

We next evaluated colitis severity by directly observing the colon in live mice by performing endoscopic analysis on the last day of the experiment (Figure 4A). The colon of mice in the healthy group showed clear vascular patterns and smooth surfaces with no signs of colitis, such as diarrhea, bleeding, or the presence of fibrin. However, the colon of mice in the untreated colitis, CsAc, and CsAc-AMP-treated groups exhibited damage such as a rough surface of the colon wall, bleeding, diarrhea, and visible fibrin. In contrast, the colon of the CsAc-EAMP-treated group showed less damage than the untreated colitis group, and only minimal signs of colitis were observed. Owing to the recovered colon wall, vascular patterns similar to those in the healthy colon were observed. For the quantitative evaluation of colitis severity using endoscopy, MEICS scores were calculated (Figure 4B). The untreated colitis, CsAc, and CsAc-AMP-treated groups showed significantly elevated MEICS values compared with the healthy group due to the presence of various signs of colitis. Unlike other CsA formulations, treatment with CsAc-EAMPs relieved inflammatory reactions, followed by a decrease in MEICS values. The endoscopic results revealed that CsAc-EAMPs could efficiently promote colonic tissue recovery following inflammation.

#### 3.3.3. Histological Analysis of Colitis 

**Figure 5:**
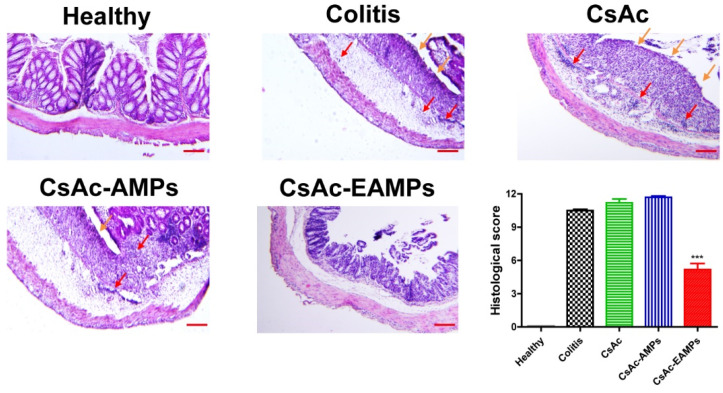
Representative histological images and histological scores of the colon, treated with or without CsA formulations. Orange and red arrows indicate damaged epithelium and infiltrated immune cells, respectively. Scale bar represents 200 µm. *** denotes *p* < 0.001 compared with the untreated colitis group. Data are expressed as means ± standard deviation (*n* = 3). CsAc, cyclosporine A crystals; CsAc-AMPs, CsA crystal-loaded alginate microparticles; CsAc-EAMPs, CsA crystal-loaded, Eudragit S 100 coated alginate microparticles.

To further investigate the enhanced anti-inflammatory effects of CsAc-EAMPs, colon tissue samples were histologically assessed by microscopic examination of H&E-stained sections (Figure 5). In the healthy group, the colonic tissue showed no signs of inflammation or epithelial layer disruption. However, tissue samples from the untreated colitis, CsAc- and CsAc-AMP-treated groups showed epithelial layer destruction, edema, and marked infiltration of inflammatory cells into the lamina propria, indicating inflammatory reaction and unhealed tissue damage. Accordingly, the histological scores of these groups were significantly higher than those of the healthy group. In contrast, colon tissues from mice treated with CsAc-EAMPs displayed a more well-differentiated morphology than the untreated colitis group, and immune cell infiltration into the lamina propria was not observed due to reduced colonic inflammation. In addition, the histological score of the CsAc-EAMPs-treated group was significantly lower than that of the untreated colitis group; however, the score was higher than that of the healthy group, given the remaining damage in affected tissue samples of the CsAc-EAMP-treated group. Collectively, the histological analysis revealed that CsAc-EAMP treatment could effectively promote the healing of damaged tissues by alleviating inflammatory reactions in the colon.

#### 3.3.4. Immunostaining of E-Cadherin

**Figure 6:**
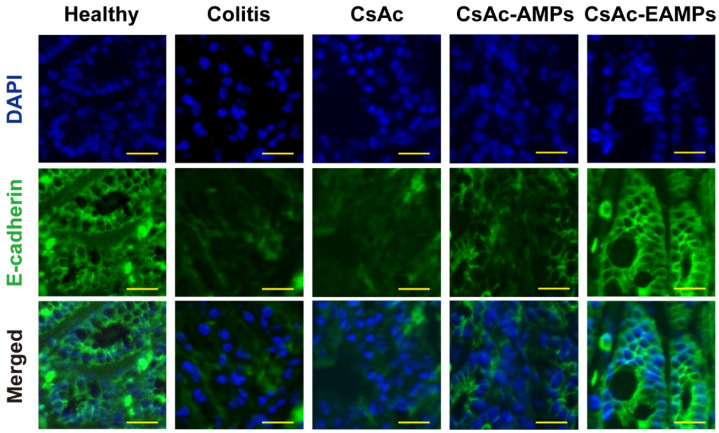
Representative CLSM images of tissue sections subjected to E-cadherin immunostaining. Scale bar represents 100 µm. CsAc, cyclosporine A crystals; CsAc-AMPs, CsA crystal-loaded alginate microparticles; CsAc-EAMPs, CsA crystal-loaded, Eudragit S 100 coated alginate microparticles; CLSM, confocal laser scanning microscopy.

The severity of colitis in colon tissue samples treated with or without CsAc formulations was further evaluated by E-cadherin immunostaining (Figure 6). As E-cadherin plays a key role in epithelial cell-to-cell adhesion and maintenance of intestinal barrier function [40], recovery of E-cadherin expression is a critical indicator of mucosal healing in UC. The CLSM image of the healthy colon showed distinct green fluorescence (E-cadherin), while that of the untreated colitis group exhibited dim green fluorescence owing to the mucosal damage caused by DSS-induced severe inflammatory reactions. Similar to the untreated colitis group, CsAc- and CsAc-AMP-treated groups also showed low levels of E-cadherin expression, as colonic inflammation was not sufficiently reduced, given the limited drug accumulation in the colon. In contrast, the CsAc-EAMP-treated group exhibited stronger green fluorescence than the untreated colitis group. As CsAc-EAMP-treatment reduced inflammation in the colon, tissue recovery was facilitated, resulting in increased E-cadherin expression in the colonic epithelium. These results indicated that CsAc-EAMPs administration could prompt barrier function recovery in the colon via effective modulation of colitis.

#### 3.3.5. MPO Activity

**Figure 7:**
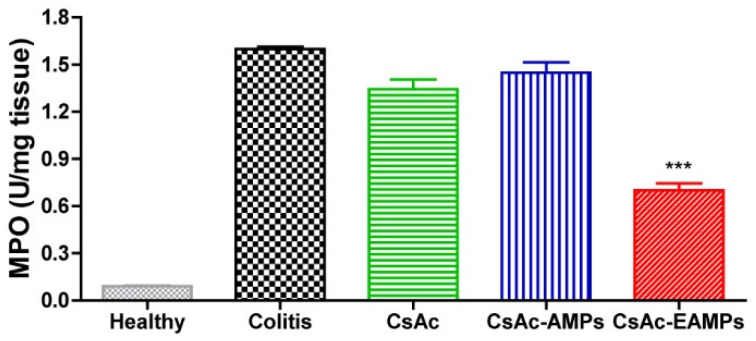
MPO activity in colon samples treated with or without CsAc formulations. *** denotes *p* < 0.001 compared with the untreated colitis group. Data are presented as means ± standard deviation (*n* = 8). CsAc, cyclosporine A crystals; CsAc-AMPs, CsA crystal-loaded alginate microparticles; CsAc-EAMPs, CsA crystal-loaded, Eudragit S 100 coated alginate microparticles; MPO, myeloperoxidase.

To confirm colonic inflammation, activity of MPO, a representative inflammatory marker, was assayed on the last day of the experiment. Figure 7 shows MPO activity in colon tissues treated with or without CsA formulations. The MPO activity was significantly higher in colon tissues isolated from the animals from untreated colitis, CsAc, and CsAc-AMP-treated groups than in the animals of healthy control group; this indicated that inflammation did not improve following treatment with CsAc and CsAc-AMPs. In contrast, MPO activity was significantly reduced in colon tissues from the CsAc-EAMP-treated group when compared with that in the untreated colitis group.

## 4. Discussion

Although CsA is a potent anti-inflammatory drug for the treatment of UC, only a limited success has been achieved due to the severe side effects resulted from the unwanted systemic distribution of CsA. For the reason, development of targeted delivery system that could minimize systemic distribution of CsA and increase drug accumulation at the target site is needed. Until now, various colon-targeted drug delivery systems including SmPill^®^ and coating techniques such as Duocoat^®^, and Colpulse^®^ have been developed for increasing drug concentration in the colon [41,42,43]. However, these delivery systems achieved limited successes in terms of a precise drug delivery to the colon; thus, development of an efficient colon-targeted system still remains challenging. In the present study, in order to deliver a large amount of CsA to the inflamed tissues in the colon without systemic absorption, CsA pure drug crystals-incorporated pH-responsive microparticular drug delivery system was successfully developed and the enhanced anti-inflammatory effects of the formulation was demonstrated using a DSS-induced colitis mouse model. After micronization of CsA powder, 3.1 ± 0.9 μm-sized homogeneous pure drug microcrystals were prepared via sonication-assisted anti-solvent precipitation (Figure 1). Then, CsAc was incorporated into the alginate microparticles and coated with ES to prevent the premature release of CsA in the stomach and small intestine. However, owing to the negatively charged CsAc-AMP surface and the negatively charged nature of ES, direct surface coating of CsAc-AMPs with ES was inefficient. Thus, before coating with ES, the CsAc-AMP surface was modified using positively charged chitosan. Subsequently, ES was successfully adsorbed on the surface of CsAc-AMPs via electrostatic interactions between the positively charged chitosan-modified surface of CsAc-AMPs and negatively charged ES. CsA incorporation into alginate microparticles did not require any organic solvent for solubilizing CsA, which is known to result in drug loss during particle preparation. Accordingly, high loading (48 ± 5%) and encapsulation efficacy (77 ± 9%) were achieved (Table 1). In this regard, one of the major limitations of the previously reported colon-targeted drug delivery systems using CsA is the low drug loading (less than 10% of CsA loading) [11]. In this study, this limitation had successfully been overcome by incorporating pure CsA crystals into the colon-targeted drug delivery system.

After successful fabrication of CsAc-EAMPs, their enteric protective effects were confirmed by examining drug release profiles in the pH-changing media that simulate the pH conditions of the GI tract. As shown in Figure 2, drug release was efficiently suppressed (˂20% of total CsA was released) under conditions simulating the stomach and small intestine (pH 1.2 for 0 to 2 h and pH 6.8 for 2 to 5 h, respectively) owing to the enteric protection afforded by ES-coated alginate microparticles. Since CsA released from CsAc-EAMPs in the stomach and small intestine can be absorbed and causes severe systemic side effects, inhibition of CsA release before reaching the colon would be beneficial not only for reducing drug loss but also minimizing off-target toxicity. Nevertheless, concerns about systemic side effects still remain in CsAc and CsAc-AMPs since more than 50% of total CsA was released under the stomach and small intestine conditions. Under the colonic pH condition (pH 7.4), the pH-responsive dissolution of ES and disintegration of alginate microparticles facilitated CsA release, and more than 80% of total CsA was released for up to 24 h in a sustained manner. The enhanced drug release phenomenon of the CsAc-EAMPs after exposure to the colonic pH condition would also be attributed to the facilitated swelling and disintegration of the alginate microparticles under the pH > 7 condition [44]. Thus, the drug release mechanisms provided by both Ca-alginate and ES would be more reliable than the delivery carriers coated only with ES because a single-layered ES coated colon delivery system may result in incomplete dissolution due to a thick ES layer, resulting in therapeutic failure. For these reasons, dual functional drug release triggering systems have emerged, and some of the advanced tablet coating technologies such as Phloral^®^ and OPTICORE™ have been successfully commercialized [45,46]. In addition to the colon-specific drug release profiles, CsAc showed enhanced accumulation in the inflamed area of the colon due to size-dependent bioadhesion effects [47]. Accordingly, CsAc-EAMPs could provide sufficient CsA molecules to the colitis region and minimize systemic side effects by restricting premature drug release before reaching the colon.

Next, the enhanced in vivo therapeutic efficacy of CsAc-EAMPs was evaluated in a DSS-induced colitis mouse model, which demonstrates characteristics similar to those of human UC [48]. Following the successful colon-targeted delivery of CsA, treatment with CsAc-EAMPs improved colitis symptoms, including body weight loss, rectal bleeding, and diarrhea, thus resulting in a reduced DAI when compared with that of the untreated colitis group (Figure 3A). Moreover, the CsAc- and CsAc-AMP-treated groups and the untreated colitis group exhibited markedly reduced colon length; however, the CsAc-EAMP-treated group showed significantly increased colon length when compared with that of the untreated colitis group (Figure 3B,C). In CsAc- and CsAc-AMP-treated groups, although 30 ~ 40% of CsA could be released in the colon, the amount of CsA in the colitis region was not enough to exert anti-inflammatory effects. Due to the low delivery efficiency of CsAc and CsAc-AMPs, there were no significant therapeutic activities in this experimental setting (15 mg/kg, daily). For example, it was reported that around 4.7 times higher dose (70 mg/kg, daily) of CsA was required to alleviate DSS-induced colitis when no delivery system was used [49]. On the other hand, in the CsAc-EAMP-treated group, since around 80% of CsA could be available in the inflamed colon, we presume that a sufficient amount of CsA can be accumulated in the colitis region resulting in facilitated alleviation of UC.

As a result of rapid colitis relief, the colon cavity, as observed endoscopically, exhibited a higher number of ameliorated features in the CsAc-EAMP-treated group than in the untreated colitis group (Figure 4). Conversely, the colons of CsAc- and CsAc-AMP-treated groups, as well as the untreated colitis group, exhibited distinct signs of colitis. In addition to rectal bleeding and diarrhea, the surface of the colon was thickened, and fibrin tissues were frequently observed during endoscopic analysis. The colon surface damage induced by severe inflammation was confirmed by histological analysis. As shown in Figure 5, CsAc- and CsAc-AMP-treated groups and the untreated colitis group showed epithelial disruption, with large numbers of immune cells observed in the lamina propria and mucosa, characteristic features of colitis. However, the epithelium showed greater recovery of morphological features in the CsAc-EAMP-treated group than the untreated colitis group, and the immune cell infiltration was not observed, indicating that the inflammatory reaction was alleviated following CsAc-EAMPs administration.

After macroscopic and endoscopic analysis, epithelium recovery in colon tissues treated with or without CsA formulations was examined by assessing E-cadherin expression in the colon epithelium. E-cadherin plays a major role in maintaining colon tissue architecture and adhesion between epithelial cells [50]. E-cadherin expression is reportedly reduced during colitis, which is linked to the disturbed intestinal barrier function and homeostasis [40]. In the present study, E-cadherin expression in the colon epithelium was markedly decreased in CsAc- and CsAc-AMP-treated groups, as well as the untreated colitis group, compared with that in the healthy group. However, CsAc-EAMP treatment significantly increased E-cadherin expression in the colon epithelium (Figure 6). As disruption of the epithelium caused by inflammatory reactions was ameliorated following CsAc-EAMP administration, epithelial recovery was facilitated, resulting in higher expression of E-cadherin than that in the untreated colitis group.

Finally, MPO activity was measured in colon tissues to evaluate the severity of inflammation (Figure 7). MPO, a representative biological marker of inflammation, is the most abundant protein in neutrophils [40]. As neutrophils play a crucial role in the pathogenesis of colitis and infiltrate the colon tissues in UC, MPO levels were dramatically increased in the CsAc- and CsAc-AMP-treated and untreated colitis groups when compared with that in the healthy group. In contrast, the CsAc-EAMP-treated group showed significantly lower MPO levels than the untreated colitis group due to reduced immune cell infiltration and inflammation in colon tissues.

Collectively, the enhanced therapeutic effects of CsAc-EAMPs were attributed to the colon-targeted delivery that enhanced CsA accumulation in the inflamed colonic regions to mitigate inflammation. Given the absence of colon-targeted delivery in the CsAc and CsAc-AMP-treated groups, CsA accumulation in the colitis area was insufficient to relieve inflammation, resulting in therapeutic failure in DSS-induced colitis mice.

## 5. Conclusions

In the present study, CsAc-EAMPs were developed for targeted delivery of CsA to enhance therapeutic efficacy and minimize systemic side effects of CsA. Herein, 47.1 ± 6.5 µm-sized CsAc-EAMPs were successfully fabricated with 48 ± 5% CsA loading and 77 ± 9% EE via three sequential processes: fabrication of CsAc, incorporation of CsAc into alginate microparticles, and ES coating on the microparticle surface. In addition, CsA release from CsAc-EAMPs was effectively suppressed under simulated pH conditions of the stomach (pH 1.2) and small intestine (pH 6.8), indicating that CsA absorption could be restricted to reduce systemic side effects. Finally, CsAc-EAMPs showed enhanced anti-inflammatory effects in a DSS-induced colitis mouse model. Collectively, these results suggest that CsAc-EAMPs could be a potent colon-targeted CsA delivery system for the treatment of UC.

## Figures and Tables

**Table 1:** Physicochemical characteristics of CsA formulations.

Formulations	Particle Size (µm)	Encapsulation Efficiency (%)	CsA Loading (%)
CsAc	3.1 ± 0.9	-	-
CsAc-AMPs	46.4 ± 8.8	78 ± 7	49 ± 9
CsAc-EAMPs	47.1 ± 6.5	77 ± 9	48 ± 5
